# Intervention Strategies to Address Barriers and Facilitators to a Healthy Lifestyle Using the Behaviour Change Wheel: A Qualitative Analysis of the Perspectives of Postpartum Women

**DOI:** 10.3390/nu16071046

**Published:** 2024-04-03

**Authors:** Siew Lim, Sarah Lang, Melissa Savaglio, Helen Skouteris, Lisa J. Moran

**Affiliations:** 1 Health Systems and Equity, Eastern Health Clinical School, Monash University, 5 Arnold Street, Boxhill, VIC 3128, Australia; 2 Monash Centre of Health Research and Implementation, Monash University, 43-51 Kanooka Grove, Clayton, VIC 3168, Australia; sarah.lang1@monash.edu (S.L.); melissa.savaglio@monash.edu (M.S.); helen.skouteris@monash.edu (H.S.); lisa.moran@monash.edu (L.J.M.); 3 School of Public Health and Preventive Medicine, Monash University, 553 St Kilda Road, Melbourne, VIC 3004, Australia

**Keywords:** behaviour change, behaviour therapy, dietary intervention, healthy lifestyle, mental health, postpartum, sleep deprivation

## Abstract

Postpartum women experience unique barriers to maintaining healthy lifestyles after birth. Theory-based behaviour change techniques and intervention strategies can be integrated into postpartum lifestyle interventions to enable women to overcome barriers to change. This study aims to explore barriers and facilitators to engaging in healthy postpartum lifestyle behaviours and develop intervention strategies for integration in a postpartum lifestyle intervention using the Behaviour Change Wheel (BCW). Semi-structured interviews were conducted with women up to two years postpartum (n = 21). Interviews were thematically analysed, themes were mapped to the Capability, Opportunity, and Motivation Model of Behaviour Change and intervention strategies were developed using the BCW. Findings suggest that women face barriers and facilitators within capability (sleep deprivation, mental exhaustion, ability to plan), opportunity (support of friends, partners and extended families) and motivation (challenges with prioritising self, exercise to cope with stress). Intervention strategies included supporting behaviour regulation and sleep to enhance capability, engaging partners, strengthening peer support to create opportunities and highlighting the mental health benefits of healthy lifestyles to inspire motivation. Integrating targeted evidence-based behaviour change strategies into postpartum lifestyle interventions may support women in overcoming commonly reported barriers to a healthy lifestyle.

## 1. Introduction

The postpartum period is a critical life stage for engaging women in healthy lifestyle behaviours for obesity prevention, reduction of future chronic disease risk and optimising health for future healthy pregnancies [1]. Postpartum weight retention is a significant contributor to obesity and weight gain in women [2], with women retaining, on average, 0.5 to 4.0 kg from each pregnancy by one year after birth [3,4]. The inability to return to pre-pregnancy weight or further weight gain in the postpartum period is associated with significant risks to maternal health, including increased risk of future cardiometabolic disease, such as cardiovascular disease and diabetes [5,6,7]. Interpregnancy weight gain is also associated with an increased risk of gestational diabetes, pre-eclampsia, caesarean delivery, stillbirth and large-for-gestational-age birth in subsequent pregnancies [8,9].

Postpartum lifestyle interventions, designed to optimise dietary intake and physical activity, have been shown to result in significant improvements in body weight, BMI and physical activity amongst postpartum women compared to minimal treatment controls [10]. However, postpartum interventions have low program penetration and participation rates [11]. Women experience significant barriers to engaging in healthy lifestyles unique to the postpartum period, including childcare needs, recovery from childbirth, tiredness and lack of time [12]. Interventions targeting lifestyle-related behaviour change in postpartum women should address these barriers while strengthening the facilitators of healthy lifestyles to optimise intervention efficacy and uptake amongst women. Women’s insights on barriers and facilitators to a healthy lifestyle unique to the postpartum period can be used to determine theory-based intervention strategies for inclusion in a lifestyle intervention [13]. Using women’s insights to inform intervention development extends the value of qualitative research beyond simply exploring barriers and facilitators to deriving practical strategies that engage and equip women with relevant knowledge, skills and opportunities to navigate and overcome barriers to change. 

Applying theory to the design and evaluation of complex behaviour change interventions is considered good practice [14], with theory-based interventions shown to be effective in promoting health-related behaviour change [15,16,17]. The Behaviour Change Wheel (BCW) is a meta-framework synthesising 19 behaviour change frameworks into a comprehensive, systematic, theory-based approach to intervention design [18]. At the centre of the BCW framework is a ‘behaviour system’ encapsulating three essential conditions to facilitate behaviour change: capability, opportunity and motivation (COM-B). The COM Model of Behaviour Change posits that one’s ability to engage in a behaviour (capability), the enabling circumstance (opportunity) and the willingness to engage (motivation) need to be present for a particular behaviour to occur [13,18]. The BCW approach to intervention development begins with understanding the problem in behavioural terms, particularly identifying opportunities for behaviour change to promote capability, opportunity and motivation [13]. These opportunities for behaviour change inform the selection of theory-based intervention functions and strategies for integration into a lifestyle intervention. This study aims to explore barriers and facilitators to engaging in a healthy postpartum lifestyle and develop intervention strategies for integration into a postpartum lifestyle intervention using the Behaviour Change Wheel.

## 2. Methods

### 2.1. Study Design

This study employs a qualitative descriptive study design to explore and document women’s experiences managing a healthy lifestyle after birth, with the epistemological foundation of study design and analysis being pragmatic. This study was conducted and reported as per the Consolidated Criteria for Reporting Qualitative Research (COREQ) guidelines (Table S1) [19]. The methodology undertaken ensures the credibility (member checking), dependability (data saturation), confirmability (documenting steps, 10% cross-checking of transcript coding) and transferability (field notes, reflexive journal, presenting interview quotes and participant characteristics) of the data [20]. This study was approved by the Monash University Human Research Ethics Committee (Project number: 22952) and Monash Health Human Research Ethics Committee (Reference number: RES-19-0000-685A).

### 2.2. Participants and Data Collection

Women who had given birth within the last two years and were living with their child were recruited via SMS, email or phone via convenience and snowball sampling. Purposive sampling was also conducted to ensure the representation of women from rural or remote areas and those from culturally and linguistically diverse backgrounds. Women <18 years, currently pregnant or unable to easily communicate in a semi-structured interview in English were ineligible. Some interviewees had prior social or professional relationships with the interviewers due to the nature of convenience sampling. Semi-structured interviews (30–40 min) were conducted via Zoom (Version: 5.4.2) to facilitate women’s engagement with young children.

Interviews were completed between 15 May 2020 to 20 July 2020. The interview schedule and interviews were developed and conducted by female research dietitians (S.Lim and LM) who are experienced in developing lifestyle interventions and conducting qualitative research. The schedule was pilot-tested in two postpartum women. The interview schedule covered participants’ perspectives of a healthy lifestyle, facilitators and barriers to a healthy lifestyle after birth and preferred forms of support to enable a healthy lifestyle after birth (Table S2). Informed written or verbal consent was obtained prior to the interview. Participants knew that researchers were conducting a series of research studies on optimising women’s postpartum health. Participants were encouraged to find a comfortable, quiet space for the interview, such as their home or office. Other family members, such as partners and children, may have been present but did not participate in the interview. No other researchers were present during the interviews.

Interviews were conducted until data saturation occurred, which was defined as no new emerging themes from the participants’ responses. Interviews were audio-recorded and transcribed using a professional transcription service. Interview transcripts were sent to participants for verification (i.e., member checking). Demographic information was collected from interview participants through an online survey distributed via Qualtrics prior to the interview, including age, date of recent birth (i.e., postpartum age), complications during pregnancy (i.e., gestational diabetes), pre-pregnancy body mass index, gestational weight gain, breastfeeding status, employment, country of birth and education.

### 2.3. Data Analysis

Participant characteristics were analysed using Microsoft Excel (Microsoft Office Professional Plus 2016). Categorical data were reported as frequencies, while continuous data were reported as means and standard deviations. Interview transcripts were analysed thematically using a template analysis approach as outlined by King [21] and Brooks et al. [22]. Following familiarisation with the dataset, a subset of transcripts (10%) were inductively coded by two authors (MS [10%], S.Lim [5%], LM [5%]). Three authors discussed and deliberated initial codes (MS, S.Lim, LM). Codes were organised into meaningful clusters to develop a preliminary coding template with initial integrative themes. The coding template was applied to all remaining transcripts by one author (MS) and iteratively updated as required. The coding template was finalised and interpreted to develop the final themes in discussion with the authorship team. The main themes were subsequently mapped to the COM-B domains (Table S3). Direct quotations from the transcripts are used to illustrate key themes. Pseudonyms are used to maintain participant anonymity. Data were managed using a qualitative data analysis software program (NVivo 12; QSR International 1999–2018).

### 2.4. Development of Intervention Strategies

The BCW Guide to Designing Interventions informed the development of intervention strategies for inclusion in a postpartum healthy lifestyle intervention [13]. Initially, opportunities for behaviour change were identified using the facilitators and barriers identified during the thematic analysis. The ‘target’ behaviours for behaviour change were then mapped to relevant domains within the COM-B and Theoretical Domains Framework (TDF) [13]. Relevant intervention functions, or “means by which an intervention can change behaviour” [13], were selected according to the TDF domain of each ‘target’ behaviour (Table S4) as per the BCW guide [13,18]. Behaviour change techniques (BCTs) were informed by the Behaviour Change Technique Taxonomy [23]. BCTs associated with each intervention function (Table S5) as per the BCW guide were listed. Intervention strategies were then iteratively developed by one author (S.Lang) in response to each identified BCT relating to the ‘target’ behaviour. The APEASE criteria (affordability, practicability, effectiveness/cost-effectiveness, acceptability, side-effects/safety, equity) were used to make strategic judgements on the appropriateness of BCTs and associated intervention strategies for integration into a postpartum healthy lifestyle intervention. BCTs and intervention strategies that did not meet the APEASE criteria are listed. Proposed intervention strategies were refined with the research team (S.Lim, S.Lang, LM).

## 3. Results

Interviews with postpartum women (n = 21) ranged from 26 to 47 min. One additional woman who expressed interest was subsequently lost to contact. Women were between 30–44 years with the majority of women born in Australia (n = 13), tertiary educated (n = 20) and in part-time or full-time paid employment (n = 19) (Table 1). Two women were involved in voluntary or unpaid work outside the home. A subset of women had experienced gestational diabetes (n = 4) or pre-eclampsia (n = 5) during their most recent pregnancy, had a pre-pregnancy BMI of 25 kg/m^2^ or above (n = 5) or were currently breastfeeding (n = 10). Self-reported gestational weight gain ranged from 6 kg to 28 kg. Barriers, facilitators and key themes relating to each domain (capability, opportunity and motivation) of the COM model were identified. See Table S3 for additional exemplary quotes for each theme.

### 3.1. Defining a Healthy Lifestyle

Women defined health and a healthy lifestyle in a holistic and integrated manner that extended beyond solely eating well and being active. Women considered a healthy lifestyle multifaceted, encapsulating physical, social, mental and emotional health. Eating well, being active, having good quality sleep and feeling rested and energetic were important. A healthy mind, stress management and the associated sense of calm, happiness and positivity were critical components of health. Positive relationships with family and friends enabled feelings of connection. A healthy lifestyle was often described as when all these domains were achieved and in “balance”. Women drew on this definition when describing personal facilitators and barriers to a healthy lifestyle.

*“[My son] last week he looked at me and he asked me why I’m not laughing as much. … I think that, like everything that has been going on, it’s been affecting him in some way …. So, a healthy lifestyle for me is mainly being happy.”*

(Alice, 15 months postpartum)

*“I think it [a healthy lifestyle] is a balance between looking after yourself mentally, and physically, and emotionally.”*

(Patricia, 1 month postpartum)

### 3.2. Barriers to Healthy Lifestyle in the Postpartum Period

Women expressed numerous barriers relating to capability, opportunity and motivation that prevented them from engaging in healthy lifestyle behaviours (Table 2).

#### 3.2.1. Mentally Exhausted: Lack of Sleep and Mental Burden

Women faced significant barriers to keeping themselves healthy relating to their psychological *capability*. Sleep deprivation and mental burden were commonly cited barriers to a healthy lifestyle. Interrupted sleep at night and a continuous list of scheduled daily tasks, including infant care, home duties and paid work, resulted in physical and mental exhaustion and a lack of headspace to plan and engage in healthy lifestyle behaviours.

*“By the end of the day, I’m pretty exhausted to, to cook a meal or to do anything really, (laughs) I just want to lie on the couch.”*

(Farah, 13 months postpartum)

*“Sometimes your mental, I mean, your brain space is only this many, and your concentration is, can be, you know, it’s limited. And there are many areas that you need to look after.”*

(Mia, 2 months postpartum)

Some women also commented on the reduced quality of sleep, such as sleeping with ‘one eye open’ (Farah, 13 months postpartum) or having ‘insomnia’ (Patricia, 1 month postpartum), resulting in low energy levels throughout the day. Sleep issues were intertwined with mental health concerns. Stress affected sleep in some women, while sleep deprivation resulted in anxiety and poor mental or emotional health in others. One woman also cited that tiredness led to more frequent snacking.

#### 3.2.2. Time Poor: Too Busy to Be Healthy

Women after birth may have reduced *opportunity* to engage in healthy lifestyle behaviours, with the most cited barrier being lack of time. Women described being constantly busy, resulting in not having time to attend health programs, exercise and sometimes even eat.

*“You’re so busy when you’ve got a baby and when it’s not your first baby you’re even busier.”*

(Phillipa, 19 months postpartum)

Finding a continuous period of time to exercise was often challenging, with one woman citing “three minutes is probably the max[imum]” (Frances, 13 months postpartum) time she could get a break from her baby. Having older siblings in addition to the newborn baby added to the busyness. Most discussions around lack of time related to the inability to exercise. However, several women described how a lack of time impacted their eating patterns, such as having only one proper meal a day to suit their busy schedules. 

#### 3.2.3. Unsupported: Lack of Practical and Social Support from Partner, Family and Friends

The *opportunity* women have to engage in healthy lifestyle behaviours was further impeded by the lack of practical and moral support from their partner, family and/or friends. Most women described their husbands being busy with work as a key barrier to a healthy lifestyle. Responsibilities around food preparation were usually not equally shared between partners, with household responsibility often primarily falling on women. Some women also cited an inability to share infant care with their partners.

*“During the week unfortunately he leaves at, you know, quarter to seven and he doesn’t get home till 7:00 pm. So, the responsibility for the lifestyle, you know, the food choices, are purely mine.”*

(Adele, 23 months postpartum)

*“I couldn’t really leave her with (my partner), she was always with me all the time.”*

(Maeve, 1 month postpartum)

Furthermore, several women described not having extended family living in the same city as missing an important source of support. The community parents’ group offered by local councils only involved first-time mothers, thus mothers of subsequent births were not invited to these services. All of this culminated in an isolating experience of motherhood for some.

*“Having kids in Australia is a very lonely process for the first year if you don’t have anybody helping you.”*

(Hua, 19 months postpartum)

#### 3.2.4. A Balancing Act: Difficulty Prioritising Self in the Midst of Competing Priorities

In terms of *motivation,* women expressed that they prioritised their children, husband, work and even pets before themselves. Prioritising their newborn’s needs was most frequently discussed.

*“You always come last, not first, and work comes second, and then, you know, family and friends and other things, and then you’re kind of, the last to kinda get looked after in that process.”*

(Emma, 18 months postpartum)

There was a sense that this was a temporary and unique phase in life that warranted a pause in women’s self-care to focus on the baby. Women postponed their intention for self-care by expressing that they could have more time for themselves again later in life. Engaging in self-care was sometimes described as “impossible” if it involved leaving the baby.

*“For this time, it’s his time, and then when he’s older, then I’ll have my time again.”*

(Patricia, 1 month postpartum)

### 3.3. Facilitators to Healthy Lifestyle in the Postpartum Period

Women also identified facilitators that allowed them to engage in healthy lifestyle behaviours, particularly eating healthily and being active (Table 2). These facilitators are described in the main themes below.

#### 3.3.1. Fitting It in: Organise and Plan Ahead

Some women demonstrated extraordinary capability to overcome the constraints of lack of time and busyness by being organised and planning. Identifying short periods, such as the baby’s nap time, to engage in physical activity and integrating physical activity into daily routines were strategies used to navigate time constraints. A regular weekly routine for household tasks, infant care and exercise was described as helpful. Planning ahead often reflected an intention to prioritise healthy behaviours.

*“I think that for me trying to sort of focus on maintaining, you know, health and wellbeing and also just a bit of happiness and, you know, that, a routine is quite important, so I’m probably maybe sometimes a little bit too rigid just on myself, but I, it makes me feel better knowing, okay, Mondays I do this, Tuesdays, I do this. You know, etc., etc., because then I... let’s me sort of be organized and prepared and help shape the week.”*

(Kate, 17 months postpartum)

*“Sometimes I try to squeeze in 15 min, um, just on my bicycle at home, before I pick up [child]. … I reckon if its, if I can squeeze in, three 15-min bicycle sessions a week, during the week days, that’s very good. Of course, on weekends I try to do a bit more.”*

(Cheng, 23 months postpartum)

Women often justified self-care activities relating to sleep, diet, physical activity and mental wellbeing as important and beneficial for themselves and their families. Several women expressed intentions and used plans and routines to create restful time for themselves to prioritise and maintain good mental wellbeing.

*“You really need to take care of your mental health and you need to make sure that you, you have your space”*

(Farah, 13 months postpartum)

*“When you’re doing the exercise, you do find then that sometimes things will become pretty obvious to me that they weren’t before. So, for example, I find if I go swimming or something, suddenly all these jobs that I had to do become quite clear, and it’s almost like an orderly list in my mind.”*

(Phillipa, 19 months postpartum)

#### 3.3.2. Friends for Health: The Importance of Other Mothers

Support from other women was among the most cited facilitator in enabling healthy behaviour regarding *opportunity*. Peers provided acknowledgement, validated their experiences and shared solutions to challenges of staying healthy with a newborn. Peers were reported to inspire healthy lifestyle behaviours through modelling, accountability and encouragement, alongside providing other social benefits of friendship. Mothers’ groups were sometimes the setting for this peer support, while other sources include church friends, social media and exercise buddies. Face-to-face events with friends and group messages enabled access to advice and support from other mothers.

*“They were one of them …that time was very cold days and she still went out for a walk. I don’t know where she, where they went out for, like to nature walk and they actually saw kangaroos…it reminded me of that time after, the feel after I exercise”*

(Olivia, 8 months postpartum)

*“Just as someone to talk to and share and relate to, that’s really, a really nice support.”*

(Kate, 17 months postpartum)

#### 3.3.3. The Better Half: Partners as Source of Practical and Moral Support for Health

Apart from friends, support from partners was also greatly valuable in facilitating self-care in women after birth, creating further opportunity for healthy lifestyle behaviours. When partners shared the burden of childcare and household chores, women could engage in exercise or self-care during that time.

*“He was very supportive of me going to the gym. So, he’ll make time for me so I can go there for an hour or two for exercise.”*

(Hua, 19 months postpartum)

Apart from offering practical help, partners could also be a form of social or moral support. Several women described that it was important to have their partners “on the same page” to prioritise a healthy lifestyle.

*“A husband that shares the same value. When he sees that I keep up with my exercise, he doesn’t want to be on the loser end as well, because he tries to keep up with me, then when I see that, “Oh, he’s doing his. I better do mine too.”*

(Olivia, 8 months postpartum)

*“The fact that [my husband] is sort of a driving force for us having a healthy lifestyle is very helpful. I think that both, both parents have to sort of be on the same page”*

(Emma, 18 months postpartum)

#### 3.3.4. It Takes a Village: Practical Help from Extended Family

Practical help offered by extended family, such as sisters and grandparents, created further *opportunities* for women to engage in self-care. This help mainly took the form of babysitting but occasionally involved the provision of cooked meals, helping women to rest, exercise or reconnect with their partners.

*“My mom will come around early in the morning while the girls are still asleep, so that I can exercise”*

(Emma, 18 months postpartum)

#### 3.3.5. Reasons to Engage: Motivators for Healthy Behaviours

Women described that one of the main motivations to engage in exercise was for enjoyment and to improve their mental wellbeing. Exercise helped to alleviate symptoms of postnatal depression, clear the mind and “feel…more normal” (Cheng, 23 months postpartum).

*“Helped me with my postnatal depression as well... I think going to the gym really helped.”*

(Hua, 19 months postpartum)

Within *motivation*, some women also reported positive self-talk, such as reminding themselves that they will feel better after exercising. Setting smaller but realistic goals and exercising for enjoyment also increased women’s motivation.

***“*** *You’ve got to push yourself hard sometimes to keep doing it.”*

(Cara, 21 months postpartum)

*“Can I do it four days this week? Or can I make sure I’ve done two of those in a walk or, you know, can I try not to drink for, you know, six days or, you know, just drink on the weekends or whatever it is. I think that’s really powerful.”*

(Phillipa, 19 months postpartum)

### 3.4. The Unique Challenges of Maintaining a Healthy Lifestyle during the COVID-19 Pandemic 

Interviews were conducted in the initial months of COVID-19-related lockdowns, which presented unique barriers and facilitators to engaging in healthy lifestyle behaviours. COVID-related lockdowns were associated with increased uncertainty and stress amongst women, impacting women’s capability to engage in healthy lifestyle behaviours. Home-schooling older children placed increased demands on women’s time. Women reflected on the challenges of reduced exercise with limited opportunity to leave the house and reduced access to gyms or sporting facilities.

*“It is much harder to create opportunities for movement when you’re only allowed out for short periods a day.”*

(Phillipa, 19 months postpartum)

Nonetheless, COVID lockdowns created opportunities for a healthy lifestyle. Spending more time at home and working at home (minimising commute time) increased available time to cook healthy meals. The pandemic increased awareness of and access to food delivery services within their local community, including online grocery shopping, fruit and vegetable delivery, healthy pre-prepared meals and meal preparation kits. Online exercise videos/mobile apps were used to support exercise at home. Partners spending more time at home meant they could share childcare responsibilities.

*“Since COVID happened and the gym shut … I found online [training sessions] quite useful… I can do it when Kate [my daughter] is asleep.”*

(Adele, 23 months postpartum)

*“He [my husband] lost a little bit of work. So, he is at home... a bit more with my son. He’s gained confidence and skills, and is now more able to help more.”*

(Patricia, 1 month postpartum)

### 3.5. Development of Intervention Strategies According to the Behaviour Change Wheel

Facilitators and barriers reported by participants were used to identify opportunities for behaviour change to address commonly reported barriers to change. The target behaviours for optimising women’s capability included developing healthy sleep behaviours, practising self-care to prevent cognitive overload, developing health-promoting routines and planning a healthy lifestyle. Attaining practical and moral support from partners, extended family and peers could be addressed in a healthy lifestyle intervention to increase women’s opportunity to engage in lifestyle change. Encouraging women to be physically active for stress management, prioritising their health within the family unit and setting achievable goals were target behaviours to inspire motivation. Behaviours that did not meet the APEASE criteria included advocating for access to community mothers’ groups, or concerns related to finances or environments that were not supportive of healthy food and activity choices. Intervention strategies that could be included in a healthy lifestyle program were developed to address the above opportunities for behaviour change and used evidence-based behaviour change techniques (Table 3).

## 4. Discussion

This study described barriers and facilitators to achieving a healthy lifestyle unique to the postpartum period. We explored the needs of women after birth and used a theory-based approach to develop intervention strategies to address these commonly reported barriers to healthy lifestyle change [18]. There is the potential to integrate intervention strategies, informed by behaviour change techniques, into research interventions or clinical care to address factors affecting women’s capability, opportunity and motivation to implement healthy lifestyle behaviours. Researchers and clinicians are presented with a plethora of potential intervention strategies, which can be tailored and refined to meet the context-specific needs of their intervention or service to help optimise women’s engagement and success with lifestyle-related behaviour change in the postpartum period.

### 4.1. Increasing Capability: Low Intensity Intervention Focussing on Behaviour Regulation

Intervention strategies that are cognisant of and address women’s lack of time and busyness may promote engagement with lifestyle interventions and successful behaviour change. Postpartum women [24,25] and women of reproductive age [26] commonly report a lack of time and busyness as a barrier to maintaining a healthy lifestyle. Mental exhaustion from meeting the demands of infants, other children, partners and work were commonly reported to influence women’s capability to implement lifestyle changes. Sleep deprivation in the postpartum period further limits women’s capacity to develop routines, plan and problem-solve challenges relating to maintaining a healthy lifestyle [24]. As such, lower-intensity interventions as opposed to highly structured and prescriptive lifestyle programs with detailed plans to achieve a specified nutritional goal (e.g., 30% protein) may be more appropriate for postpartum women. Providing women with the opportunity to self-select goals that align with their lifestyle, while providing support and training for behaviour-regulation skills, such as action planning and self-monitoring, may be beneficial in overcoming this barrier to change [27,28]. Furthermore, integrating intervention strategies that address sleep issues of mothers and babies into postpartum interventions could increase the capability of women to engage in healthy lifestyle behaviours.

### 4.2. Creating Opportunity: Promoting Partner and Peer Engagement

Intervention strategies that promote practical assistance from partners with childcare or household duties, alongside increasing moral support from partners, may increase women’s opportunity to adopt healthy lifestyles. Our findings suggest that partners’ support extends beyond solely providing practical support, with moral support and encouragement from partners who value and prioritise a healthy lifestyle being a facilitator for a healthy postpartum lifestyle. Despite partner support being commonly reported as a facilitator of lifestyle change [12], partners are rarely engaged with postpartum lifestyle interventions [10]. Meaningful involvement of partners in postpartum lifestyle interventions may be a practical approach to increase women’s opportunity to implement change. Fathers also experience challenges with mental health as they transition into fatherhood and have expressed the desire for more support on managing fatherhood and partner relationships during this time [29]. Involving partners may therefore be beneficial in promoting health within the broader family unit. Alongside partners, peers were an important source of support for women in the postpartum period. Peers shared and validated women’s experiences and role-modelled healthy behaviours with a young child. The peer support model has seen some success in a postpartum lifestyle intervention implemented through parents’ groups in Australia, which reported the highest penetration rate (86%) among all postpartum lifestyle studies in a recent systematic review [30]. These findings emphasise that integrating intervention strategies to enable and encourage peer and partner support may be highly beneficial in creating *opportunities* for a healthy postpartum lifestyle.

### 4.3. Inspire Motivation; Prioritising Lifestyle Change for Good Mental Health

Postpartum women reported prioritising the needs of their newborn and family, and many women did not consider their health a priority at this life stage. Self-efficacy, outcome expectancies and risk perception all influence the intention to engage with a behaviour [31]. Optimising risk perception through providing information about the health, social and environmental consequences, alongside increasing self-efficacy with action planning, feedback, goal setting, problem-solving and self-monitoring are all behaviour change techniques that could increase postpartum women’s motivation to prioritise their health. Women also suggested that they were driven to exercise to optimise their mental health. Communicating the holistic benefits of physical activity instead of simply focusing on physical health benefits may promote motivation to adopt healthy lifestyle behaviours.

We have explored barriers to healthy lifestyle behaviours within each COM-B domain equally. Nonetheless, the extent of the behaviour change required to overcome each barrier will be context-specific and dependent on women’s circumstances. Clinicians and researchers must select and tailor relevant intervention strategies to meet the unique needs of the postpartum women they work with. For example, when considering postpartum mothers with anxiety or postnatal depression, intervention strategies such as prioritizing time for self-care and achievable goal setting are likely to be inappropriate. Other support mechanisms, treatments and intervention strategies will be warranted. 

### 4.4. Strengths and Limitations

Collating and synthesising women’s perspectives and using these qualitative insights to develop intervention strategies using a theory-based behaviour change framework is a strength of this research. However, there are several limitations inherent to the nature of a qualitative study. As this study seeks to understand the depth and not the breadth of the issue, external generalizability is limited. Due to convenience and snowball sampling, most of the women in this study had tertiary education, which may explain the absence of a lack of knowledge as a barrier to a healthy lifestyle. While women from culturally and linguistically diverse backgrounds were included, all women were Australian, and facilitators and barriers identified may be unique to an Australian context. As per the BCW Guide to Designing Interventions, exploring policy categories to help facilitate the implementation of a postpartum healthy lifestyle intervention would be an important next step in translating findings into real-world settings and ensuring the sustainability of the intervention following implementation [13].

## 5. Conclusions

Postpartum women face varied barriers and facilitators to engaging in healthy lifestyle behaviours across the COM-B domains. Barriers and facilitators related to women’s capability (sleep deprivation and mental exhaustion, ability to plan and develop health-promoting routines), opportunity (influence and support of friends, partners and extended families) and motivation (the struggle with prioritising self and exercise to cope with stress). A plethora of intervention strategies were developed to address these barriers to change. Researchers and clinicians should tailor and integrate these strategies into postpartum lifestyle programs and clinical care to enable and equip women with the skills to overcome common barriers to achieving and maintaining a healthy lifestyle.

## Figures and Tables

**Table 1:** Participant characteristics (n = 21).

Characteristic	Mean (Range)
Age (Years)	36 (30–44)
Pre-pregnancy BMI (kg/m^2^)	24 (18–41)
Gestational weight gain (kg)	13 (6–28)
Mean time postpartum (months)	14 (1–23)
	**N (%)**
History of gestational diabetes or pre-eclampsia	5 (24)
Currently breastfeeding (exclusive or partial)	10 (48)
Born in Australia	13 (62)
Residing in a rural area	2 (10)
In paid employment or unpaid work outside home	21 (100)
Tertiary education qualifications	20 (95)

Abbreviations: BMI (Body Mass Index).

**Table 2:** Barriers and facilitators to a healthy postpartum lifestyle according to the Capability, Opportunity and Motivation (COM) domains.

COM Domain	Barriers	Facilitators	Representative Quotes
**Physical capability** e.g., physical skill, strength or stamina	Recovery from childbirth	Nil identified	***Barrier:** “I had the twins by caesarean and … then I got flown to Melbourne for two weeks after I had this aching pain. Um, so coming home from that, just the toll it took on my body, … it took me probably uh, three or four months to be able to get up, um, off the floor easily.”* (Amelia, 17 months postpartum)
**Psychological capability** e.g., knowledge, psychological skills	Sleep deprivation and fatigue Mental exhaustion, cognitive overload and poor mental health Busyness and lack of time Navigating the demands of work and motherhood Limited planning	Organising and planning ahead Integrating healthy behaviours into routine Consistent and regular routines Prioritising healthy behaviours and creating restful time or space for self	***Barrier:** “If you’re feeling sleep deprived or … you’re really stressed or you’re not getting enough time to, to do everything, I think that exercise can, um, be the last priority.”* (Sophia, 1 month postpartum) ***Facilitator:*** *“I guess fitting in around the baby timetable so, you know, they have a nap in the afternoon. So that’s when I do my school planning … And then they have a snack at about 3:30, so after, you know, four o’clock is the perfect time to get outside and exercise.”* (Amelia, 17 months postpartum)
**Physical opportunity** e.g., resources	Costs and inadequate finances Unsupportive environments for activity (including weather)	Access to community mothers’ groups Supportive environment for physical activity and healthy food choices Supportive work environment and work–life balance	***Barrier:** “When you’re at home on maternity leave and you already have a reduced salary, any additional expenses is something that you try to avoid as best you can. Like we, we barely got through, I was only on maternity leave for eight months.”* (Evelyn, 15 months postpartum) ***Facilitator:*** *“So I’m very fortunate that I live in a suburb where, um, I can walk everywhere to access services. Um, so I try and fit in a chore or, or an activity, for example, um, going to the post office or going to the chemist. Um, I will walk and I will take the long route there and back, um, just to get in like a 30-min or a 45-min walk.”* (Emma, 18 months postpartum)
**Social opportunity** e.g., interpersonal influences	Lack of practical support from partners, extended family or peers Feeling socially isolated	Modelling, accountability, encouragement and social connection with peers Practical support from partners Partners with similar health goals Practical help from extended family, i.e., assistance childcare	***Barrier:** “I wouldn’t say it’s that easy for, for me to just say, ‘Oh, I’m going to go for a jog or something. Will you look after (the baby)?’”* (Frances, 13 months postpartum) ***Facilitator:*** *“The biggest thing for me, is once you hear that you’re not the only one that’s going through all these things, then it’s okay.”* (Eloise, 14 months postpartum)
**Automatic motivation** e.g., wants, needs, emotional reactions	Nil identified	Enjoyment Exercise to improve mental health	***Facilitator:** “One of the ways I cope emotionally is to walk a lot.”* (Patricia, 1 month postpartum)
**Reflective motivation** e.g., plans, evaluations, beliefs	Difficulty prioritising self and feeling unmotivated Prioritising children’s and family’s needs.	Motivating self-talk Goal setting	***Barrier:** “My health is probably at the bottom of that list.”* (Farah, 13 months postpartum) ***Facilitator:*** *“Trying to remember that I’ll feel better once it’s done.”* (Phillipa, 19 months postpartum)

Abbreviations: COM (capability, opportunity and motivation).

**Table 3:** Intervention strategies for integration into a postpartum lifestyle intervention as informed by the Behaviour Change Wheel.

Commonly Reported Facilitators and Barriers to a Healthy Lifestyle Postpartum	Relevant TDF Domain *	Intervention Functions to Promote Behaviour Change	Behaviour Change Technique Related to Intervention Functions (Most Frequently Used Only)	Example Proposed Intervention Strategies for Inclusion in a Lifestyle Intervention to Address Commonly Reported Barriers to a Healthy Lifestyle
**CAPABILITY—Physical**				
**Barrier:** (1) Recovery from childbirth **Target/Desired Behaviours**: Encourage physical activity or exercises that support recovery from childbirth	Physical Skills	Training	Behavioural practice/rehearsal	Prompt women to practise specific exercises such as pelvic floor exercises and gentle yoga to help optimise their physical health and recovery from childbirth.
			Demonstration of the behaviour	Show women a video or demonstrate practical exercises to support physical recovery (i.e., how to strengthen your pelvic floor) following childbirth.
			Feedback on behaviour	Review reports of women’s activity in the postnatal period and provide feedback on how they can continue to optimise their physical health after birth.
			Feedback of outcome of behaviour	Measure and provide feedback on objective/subjective measures of physical health after implementing exercises to assist with postnatal recovery.
			Instruction on how to perform a behaviour	Provide instructions on practical exercises to support physical recovery (i.e., gentle yoga) following childbirth.
			Self-monitoring of behaviour	Establish a method and encourage women to self-monitor exercises to optimise physical health after childbirth (i.e., an activity diary).
**CAPABILITY—Psychological capability**				
**Barriers:** (1) Sleep deprivation and fatigue (2) Mental exhaustion/cognitive overload and poor mental health **Facilitator:** (1) Creating restful time or space for self **Target/Desired Behaviours:** (1) Promote healthy sleep behaviours to optimise sleep quality (2) Practice self-care to address/prevent cognitive overload	Memory, Attention and Decision Processes	Enablement, Environmental restructuring, Training	Action planning	Prompt women to undergo detailed planning of how they will action strategies to assist with sleep quality and/or self-care, which include consideration of context (when and where), frequency (how often), duration (how long) and intensity.
			Feedback on the behaviour	Review reports of mother/baby sleep behaviours and provide feedback on how they can change behaviours to optimise sleep quality. Review and provide advice on when/how to integrate time for themselves into their current lifestyle.
			Feedback on outcome(s) of behaviour	Measure and provide feedback on objective/subjective measures of sleep quality and mental health following behaviour change.
			Goal setting (behaviour)	Set a goal for behaviours to improve sleep quality (i.e., caffeine reduction) and self-care (i.e., allocating time to rest and exercise).
			Goal setting (outcome)	Set a goal on a positive outcome of prioritising sleep (i.e., reduced frequency of waking after commencing infant sleep training) and self-care behaviours, (i.e., reducing feelings of cognitive overload).
			Instruction on how to perform a behaviour	Provide instructions on optimising maternal sleep and simple strategies for self-care with a newborn baby/young child.
			Problem-solving	Prompt women to analyse factors influencing sleep and/or self-care behaviours and collaboratively select strategies to overcome these barriers.
			Prompts/cues	Advise women to introduce reminders to the home environment to optimise sleep quality (i.e., setting an alarm to switch off electronic devices 1 hour before bed) and prompt self-care (i.e., setting an alarm to prompt a walk after dinner).
			Review and report progress behaviour and/or outcome goal(s)	Review progress with behaviour and/or outcome goals in conjunction with women. Collectively modify, set new or reset goals.
			Restructuring the physical environment	Advise women to change the home/sleep environment to optimise sleep quality, i.e., change room temperature or install blinds.
			Self-monitoring of behaviour	Train and encourage women to self-monitor and record behaviours affecting sleep (i.e., maintaining a sleep diary) or self-care (i.e., recording meditation, mindfulness, physical activity).
			Social support (unspecified)	Advise women to engage their partner/family/friends to encourage behaviours that optimise sleep quality (i.e., going to bed earlier) or self-care (i.e., encouraging physical activity).
			Social support (practical)	Advise women to arrange practical support to assist with behaviours that optimise sleep (i.e., sharing night-time infant care duties with their partner/family) and self-care (i.e., sharing childcare responsibilities to enable physical activity).
			Other BCTs (do not meet the APEASE criteria): (1) adding objects to the environment, (2) behavioural practice/rehearsal, (3) demonstration of the behaviour	
**Barriers:** (1) Busyness and lack of time, navigating the demands of work and motherhood (2) Limited planning **Facilitator:** (1) Organising and planning ahead (2) Integrating physical activity and health-promoting behaviours into routine **Target/Desired Behaviour:** (1) Planning for a healthy lifestyle (2) Develop health-promoting routines	Behavioural Regulation	Education, Enablement, Modelling, Training	Action planning	Prompt women to undergo detailed planning of how they will implement strategies to integrate physical activity into their daily routine and/or plan. This may include consideration of context (when and where), frequency (how often), duration (how long) and intensity.
			Behavioural practice/rehearsal	Prompt women to practise filling in planning tools, i.e., menu planning.
			Demonstration of the behaviour	Show women a video demonstrating how other women have successfully integrated physical activity into their usual routine (i.e., walking with their baby) or use planning tools (i.e., menu planning and shopping lists).
			Feedback on behaviour	Monitor and provide information/evaluative feedback on total physical activity (i.e., monitor total daily steps using a pedometer and provide feedback on how to increase steps).
			Feedback on outcome(s) of the behaviour	Monitor objective/subjective fitness measures and provide feedback on changing overall fitness levels after integrating additional physical activity into their daily routine.
			Goal setting (behaviour)	Set a goal for behaviours to encourage that inclusion of physical activity into women’s routine (i.e., walking to the shops instead of driving) or planning (i.e., completing a weekly menu planning tool).
			Goal setting (outcome)	Set a goal for a positive outcome of integrating activity into routines, (i.e., improved physical fitness levels) and planning (i.e., reduced stress levels).
			Information about health consequences	Provide information on the health benefits of developing healthy routines and planning ahead, (i.e., reduced risk of future chronic disease with increased physical activity).
			Information about social and environmental consequences	Provide information on the benefits of integrating physical activity into daily routine (i.e., additional time for other activities) or planning (i.e., meal planning can reduce food waste).
			Instruction on how to perform a behaviour	Provide instruction and advice on practical ways to changes routines to incorporate physical activity and instructions on how to plan physical activity and healthy eating.
			Prompts/cues	Introduce cues to the home environment to prompt women to integrate activity into their routine (i.e., put running shoes next to the car keys to encourage walking instead of driving) and encourage planning (i.e., stick a meal-planning tool to the fridge).
			Problem-solving	Prompt women or analyse factors influencing their capacity to develop healthy routines and/or plan ahead and collaboratively select strategies to overcome these barriers.
			Review behaviour and/or outcome goal(s)	Review progress with behaviour and/or outcome goals in conjunction with women. Collectively modify, set new or reset goals.
			Self-monitoring of behaviour	Establish a method and encourage women to self-monitor and record changes to their routines and implementation of planning (i.e., use of a pedometer, activity diary or fitness app to monitor physical activity after changing routine).
			Social support (practical)	Advise women to arrange practical support to facilitate healthy routines and planning (i.e., arrange childcare to enable physical activity, assistance with meal preparation).
			Social support (unspecified)	Advise women to engage their partner/family/friends to encourage healthy routine and planning ahead (i.e., being active alongside women).
			Other BCTs (do not meet the APEASE criteria): (1) adding objects to the environment, (2) restructuring of the physical environment	
**OPPORTUNITY—Physical**				
**Barriers:** (1) Costs and inadequate finances (2) Unsupportive environments for activity (including weather) (3) Unable to access community mothers’ groups **Facilitators:** (1) Adequate finances (2) Supportive environment for physical activity and healthy food choices (3) Supportive work environment and work-life balance	Environmental Context and Resources	Enablement, Environmental restructuring, Restriction, Training	Does not meet the APEASE criteria (outside of the scope of a postpartum behaviour change intervention)	
**OPPORTUNITY—Social**				
**Barriers:** (1) Lack of practical support from partners or extended family or peers (2) Feeling socially isolated **Facilitator:** (1) Practical and moral support from partners (2) Practical assistance from extended family (3) Practical and moral support from friends/peers **Target/Desired Behaviour:** (1) Encourage practical and moral support from partner and extended family (2) Encourage and advocate for support from peers	Social Influences	Enablement, Environmental restructuring, Modelling, Restriction	Action planning	Prompt detailed planning of how women will action strategies to engage with partners and family with lifestyle change/provision to practical assistance with enabling lifestyle change.
			Adding objects to the environment	Create an online peer support network as part of the intervention to encourage the development of peer support networks (social and moral support).
			Demonstration of the behaviour	Develop a training video with practical examples demonstrating how women can effectively communicate with their families to gain more support for their lifestyle changes.
			Goal setting (behaviour)	Set a goal for behaviours to encourage partner and family engagement, i.e., set a goal to allocate household tasks to each family member for the week.
			Goal setting (outcome)	Set a goal on a positive outcome of changing behaviours, i.e., improvements to subjective measures of stress after engaging family members with lifestyle change.
			Problem-solving	Prompt women or analyse factors influencing family engagement with lifestyle change and collaboratively select strategies to overcome these barriers to engaging with partner or family with behaviour change.
			Prompts/cues	Encourage families to create a written plan outlining how they will support each other with healthy lifestyle behaviours. Advise women to stick this plan to a wall or fridge as a reminder to initiate/sustain these behaviours.
			Review behaviour and/or outcome goal(s)	Review progress with behaviour and/or outcome goals in conjunction with women. Collectively modify, set new or reset goals.
			Social support (unspecified)	Advise women to engage their partner/family/friends to encourage other family members to engage with lifestyle change or other supportive behaviours.
			Social support (practical)	Advise women to arrange practical support to assist with lifestyle change, i.e., childcare, grocery shopping and exercising with their peers.
			Other BCTs (do not meet the APEASE criteria): (1) self-monitoring of behaviour, (2) restructuring the physical environment	
**MOTIVATION—Automatic**				
**Facilitator:** (1) Enjoyment (2) Exercise to improve mental health **Target/Desired behaviour:** Physical activity and adopting healthy lifestyle behaviours for stress management and good mental health	Emotion	Coercion, Enablement Incentivisation, Modelling, Persuasion	Action planning	Prompt women to undergo detailed planning of how they will action increased physical activity to improve mental health. This may include consideration of context (when and where), frequency (how often), duration (how long) and intensity.
			Credible source	Present information from a certified healthcare professional, such as a psychologist or counsellor, on how prioritising physical activity can influence mental health.
			Information about social and environmental consequences	Provide information on the benefits of a healthy lifestyle, being physically active and maternal stress management for the family’s wellbeing (i.e., reduced maternal stress may improve family functioning).
			Information about health consequences	Provide information on the benefits of being physically active and the impact on mental health.
			Feedback on behaviour	Monitor and provide information/evaluative feedback on total physical activity (i.e., monitor total daily steps using a pedometer and provide feedback on how to increase steps).
			Feedback on outcome(s) of the behaviour	Monitor and provide feedback on specific subjective or objective measures of stress after increasing physical activity.
			Goal setting (behaviour)	Set a goal for healthy lifestyle behaviours that can help to optimise mental health (i.e., following a Mediterranean-style diet or being physically active).
			Goal setting (outcome)	Set a goal for a positive mental health-related outcome of implementing healthy lifestyle behaviours.
			Problem-solving	Prompt women or analyse factors influencing their capacity to implement healthy lifestyle behaviours and collaboratively select strategies to overcome these barriers.
			Review behaviour and/or outcome goal(s)	Review progress with behaviour and/or outcome goals in conjunction with women. Collectively modify, set new or reset goals.
			Self-monitoring of behaviour	Establish a method and encourage women to self-monitor and record changes to their dietary and physical activity behaviours, alongside mental health monitoring.
			Social support (unspecified)	Advise women to engage their partner/family/friends to encourage healthy eating and physical activity.
			Social support (practical)	Advise women to arrange practical support (i.e., arrange childcare to enable physical activity and assistance with meal preparation).
			Other BCTs (do not meet the APEASE criteria): (1) adding objects to the environment, (2) demonstration of the behaviour, (3) monitoring of behaviour by others without evidence of feedback, (4) monitoring outcome of behaviour by others without evidence of feedback, (5) restructuring the physical environment	
**MOTIVATION—Reflexive**				
**Barrier:** Mothers prioritising the needs of others (infant, children and family). **Facilitator:** Prioritising time for self-care and motivating self-talk. **Target/Desired Behaviour:** Encourage women to prioritise self-care	Social Professional Role and Identity	Education, Modelling, Persuasion	Credible source	Present information from a certified healthcare professional on how prioritising maternal health can help improve family wellbeing.
			Information about health consequences	Provide information on prioritising a healthy lifestyle for the family’s wellbeing (i.e., improved family functioning, improved health and wellbeing of children, and positive role-modelling of healthy behaviours).
			Information about social and environmental consequences	Provide information on prioritising a healthy lifestyle for the family’s wellbeing (i.e., improved family functioning, improved health and wellbeing of children, positive role-modelling of healthy behaviours).
			Prompts/cues	Advise women to introduce reminders to engage in restful activities and prioritise their health (i.e., setting alarms to go for a walk).
			Self-monitoring of behaviour	Establish a method with women to monitor and record their behaviour, i.e., document the type and frequency of activities spent on prioritising their wellbeing.
			Other BCT’s (do not meet the APEASE criteria): (1) Feedback on behaviour, (2) Feedback on outcome of the behaviour, (3) Demonstration of the behaviour,	
**Facilitator:** Setting small, achievable goals. **Target/Desired Behaviour:** Setting achievable goals	Goals	Coercion, Education, Enablement, Modelling, Persuasion, Incentivisation,	Action planning	Prompt women to undergo detailed planning of how they will set and work towards achieving goals. This may include consideration of context (when and where), frequency (how often), duration (how long) and intensity.
			Credible source	Present information from a certified healthcare professional on how working towards SMART goals can facilitate behaviour change and improve wellbeing.
			Feedback on behaviour	Monitor and provide information/evaluative feedback on goal-setting skills.
			Feedback on outcome(s) of the behaviour	Monitor and provide feedback on the outcome of setting SMART goals (i.e., monitor changes to total steps with a pedometer after developing and implementing a SMART goal related to increased physical activity).
			Goal setting (behaviour)	Set SMART goals to optimise health-related behaviours.
			Goal setting (outcome)	Set a goal for a positive outcome from setting and working towards SMART goals (i.e., weight loss, improved mental health).
			Information about health consequences	Provide information on the benefits of goal setting for health (i.e., goal setting can facilitate diet/activity behaviour change to optimise health).
			Problem solving	Prompt women or analyse factors influencing their capacity to develop and implement SMART goals and/or plan ahead and collaboratively select strategies to overcome these barriers.
			Prompts/cues	Advise women to display their goals (i.e., encourage them to display a printed copy of their goals on their bathroom mirror to review, reflect on and plan how they will implement their goals whilst brushing their teeth).
			Review behaviour and/or outcome goal(s)	Review progress with behaviour and/or outcome goals in conjunction with women. Collectively modify, set new or reset goals.
			Self-monitoring of behaviour	Establish a method with women to monitor and record their behaviour (i.e., review and record adherence to SMART goals in a diary).
			Social support (unspecified)	Advise women to engage their partner/family/friends to encourage adherence to SMART goals (i.e., partner to provide moral support, such as encouragement and problem-solving).
			Social support (practical)	Advise women to arrange practical support to assist with developing and implementing their SMART goal (i.e., arrange for a family member to provide childcare to enable implementation of the goal).
			Other BCTs (do not meet the APEASE criteria): (1) adding objects to the environment, (2) demonstration of the behaviour, (3) information about social and environmental consequences, (4) monitoring of behaviour by others without evidence of feedback, (5) monitoring outcome of behaviour by others without evidence of feedback, (6) restructuring the physical environment	
**Facilitator:** Motivated to stay active to improve mental health **Target/Desired Behaviour:** Physical activity and adopting healthy lifestyle behaviours for stress management and good mental health	Beliefs about consequences	Education, Persuasion, Modelling	See section ‘Automatic Motivation—Emotion’ for relevant BCTs and proposed intervention strategies	

Table Legend: * No facilitators and barriers relating to TDF domains Beliefs About Capabilities, Cognitive and Interpersonal Skills, Intentions, Optimism, Knowledge and Reinforcement were identified. Abbreviations: BCT (Behaviour Change Technique), SMART (Specific, Measurable, Achievable, Realistic, Timely).

## Data Availability

The datasets presented in this article are not readily available as data sharing may compromise participant anonymity. Requests to access the datasets should be directed to the corresponding author.

## Supplementary Materials

The following are available online at https://www.mdpi.com/article/10.3390/nu16071046/s1, Table S1: COREQ Checklist [19], Table S2: Interview Schedule, Table S3: Qualitative Codebook and Exemplar Quotes, Table S4: Mapping of Capability, Opportunity and Motivation (COM) behaviour model domains to intervention functions as per Michie, Atkins and West [13], Table S5: Mapping of intervention functions to behaviour change techniques as per Michie, Atkins and West [13].
