# Supplementary material for: Intervention Strategies to Address Barriers and Facilitators to a Healthy Lifestyle Using the Behaviour Change Wheel: A Qualitative Analysis of the Perspectives of Postpartum Women
Source: Nutrients. 2024 Apr 3;16(7):1046. doi: 10.3390/nu16071046 (PMC11013589; doi:10.3390/nu16071046)
Supplement: Supplementary file 1 [file nutrients-16-01046-s001.zip › nutrients-2887934-supplementary.pdf]

nutrients

MDPI

Intervention Strategies to Address Barriers and Facilitators to a Healthy Lifestyle Using the Behaviour Change Wheel: A Qualitative Analysis of the Perspectives of Postpartum Women

Siew Lim [1,2,*], Sarah Lang [2], Melissa Savaglio [2,3], Helen Skouteris [2,3] and Lisa J. Moran [2]

[1] Health Systems and Equity, Eastern Health Clinical School, Monash University, 5 Arnold Street, Boxhill, VIC 3128, Australia
[2] Monash Centre of Health Research and Implementation, Monash University, 43-51 Kanooka Grove, Clayton, VIC 3168, Australia; sarah.lang1@monash.edu (S.L.); melissa.savaglio@monash.edu (M.S.); helen.skouteris@monash.edu (H.S.); lisa.moran@monash.edu (L.J.M.)
[3] School of Public Health and Preventive Medicine, Monash University, 553 St Kilda Road, Melbourne, VIC 3004, Australia
* Correspondence: siew.lim1@monash.edu

Table of Contents

Table S1: COREQ Checklist [19] 2

Table S2: Interview Schedule 3

Table S3. Qualitative Codebook and Exemplar Quotes 4

Table S4. Mapping of Capability, Opportunity and Motivation (COM) behaviour model domains to intervention functions as per Michie, Atkins and West [13] 11

Table S5: Mapping of intervention functions to behaviour change techniques as per Michie, Atkins and West [13] 12

REFERENCES 12

Table S1. COREQ Checklist [1].

No. Item	Guide questions/description	Reported on Page #
**Domain 1: Research team and reflexivity**		
Personal Characteristics		
1. Interviewer/facilitator	Which author/s conducted the interview or focus group?	Page 3
2. Credentials	What were the researcher's credentials? E.g. PhD, MD	Page 1 and Page 3
3. Occupation	What was their occupation at the time of the study?	Page 3
4. Gender	Was the researcher male or female?	Page 3
5. Experience and training	What experience or training did the researcher have?	Page 3
Relationship with participants		
6. Relationship established	Was a relationship established prior to study commencement?	Page 3
7. Participant knowledge of the interviewer	What did the participants know about the researcher? e.g. personal goals, reasons for doing the research	Page 3 and 4
8. Interviewer characteristics	What characteristics were reported about the interviewer/facilitator? e.g. Bias, assumptions, reasons and interests in the research topic	Page 3
**Domain 2: study design**		
Theoretical framework		
9. Methodological orientation and Theory	What methodological orientation was stated to underpin the study? e.g. grounded theory, discourse analysis, ethnography, phenomenology, content analysis	Page 2
Participant selection		
10. Sampling	How were participants selected? e.g. purposive, convenience, consecutive, snowball	Page 2
11. Method of approach	How were participants approached? e.g. face-to-face, telephone, mail, email	Page 2
12. Sample size	How many participants were in the study?	Page 4
13. Non-participation	How many people refused to participate or dropped out? Reasons?	Page 4
Setting		
14. Setting of data collection	Where was the data collected? e.g. home, clinic, workplace	Page 3
15. Presence of non-participants	Was anyone else present besides the participants and researchers?	Page 3
16. Description of sample	What are the important characteristics of the sample? e.g. demographic data, date	Table 1, Page 4
Data collection		
17. Interview guide	Were questions, prompts, guides provided by the authors? Was it pilot tested?	Table S2, Supplementary File Page 4
18. Repeat interviews	Were repeat inter views carried out? If yes, how many?	N/A
19. Audio/visual recording	Did the research use audio or visual recording to collect the data?	Page 3
20. Field notes	Were field notes made during and/or after the interview or focus group?	Page 2
21. Duration	What was the duration of the interviews or focus group?	Page 4
22. Data saturation	Was data saturation discussed?	Page 3
23. Transcripts returned	Were transcripts returned to participants for comment and/or correction?	Page 3
**Domain 3: analysis and findings**		
Data analysis		
24. Number of data coders	How many data coders coded the data?	Page 3
25. Description of the coding tree	Did authors provide a description of the coding tree?	Table S3, Supplementary File Page 5
26. Derivation of themes	Were themes identified in advance or derived from the data?	Page 3
27. Software	What software, if applicable, was used to manage the data?	Page 3
28. Participant checking	Did participants provide feedback on the findings?	Not completed
Reporting		
29. Quotations presented	Were participant quotations presented to illustrate the themes/findings? Was each quotation identified? e.g. participant number	Table S3, Supplementary File Page 5
30. Data and findings consistent	Was there consistency between the data presented and the findings?	Table S3, Supplementary File Page 5
31. Clarity of major themes	Were major themes clearly presented in the findings?	Page 4 - 9
32. Clarity of minor themes	Is there a description of diverse cases or discussion of minor themes?	Table S3, Supplementary File Page 5

Table S2. Interview Schedule.

Question	Follow-up question
How long have you lived here?	Who else do you live with? How many children to you have? How old are they?
Were you born in Australia?	How long since you've moved to Australia? Did you speak English before?
We are studying about healthy lifestyle but that means different things to different people. What do you think a healthy lifestyle is?	How about diet/exercise/sleep/stress management?
What is your greatest health concern now?	What effect, if any, lifestyle might have on your health?
What is your greatest health concern for your child at the moment?	What effect, if any, your lifestyle might have on your child's/children's health?
What are some of the things you are doing now that contribute to a healthy lifestyle for yourself?	How about diet/exercise/sleep/stress management?
You mentioned some of the things you are doing for yourself to have healthy lifestyle, what helps you do these things?	What about friends/family/work arrangement/finance/health professionals/mothers' group
What are some of the things that prevent you from having a healthy lifestyle?	How about infant care/breastfeeding/friends/family/work/finances?
Were you able to overcome these barriers, if so how?	How about infant care/breastfeeding/friends/family/work/finances?
How does your partner's involvement in parenting affect your ability to manage your lifestyle?	Can you tell me about how it affects your lifestyle?
Who are the best people to support you in having healthy lifestyle?	How about family/friends/health professionals/community?
What do you need most to improve your lifestyle?	How about health information for mum or baby; skills on setting goals, plans; strategies, ideas?
Where would you prefer to attend this service?	How about general practitioner/maternal child health nurse/online/community setting/allied health professionals (dietitian, exercise physiologist, psychologist, physiotherapist)
Thinking back from the time you gave birth, when is the best time to start having this support?	Would you prefer to receive lifestyle support soon after birth, or a bit later?
How intensely would you like to be supported?	How long would you like to be supported for? How often would you like to receive this support?
How willing are you to pay for this service?	Would paying help motivate you?
If anything is possible, what services / information/ resources would help postpartum women to have healthier lifestyles?	What are your thoughts on mum's coaching each other? Would you be interested to be involved if given the opportunity? Would you expect to be paid?

Table S3. Qualitative Codebook and Exemplar Quotes.

COM-B Domain	Synthesised Code	Exemplar quotes
	**THEME 1: Defining a healthy lifestyle**	
Not Applicable	A wholistic approach to health	• "When I think of a healthy lifestyle, I think, um, of eating healthy food, like well-balanced food, uh, diet and I think, uh, getting exercise is helpful to a healthy lifestyle. Um, and also, um, social and emotional, so their connections with people, um, is important." ***(Amelia, 17 months postpartum)***<br>• "When I think of a healthy lifestyle, you look at … the whole big picture of life, so healthy, healthy relationships, healthy eating… general health…. Say if I think about a healthy lifestyle, I think it encompasses all of those aspects." ***(Charlotte, 18 months postpartum)***<br>• "I guess thinking about everything that contributes to health, so obviously … health and nutrition, physical activity, or sleep … And I guess self-care, down time, um, socializing… All of those things together… And I guess also having, kind of, kind of work or activities that are, um, fulfilling as well … all would come combined, for me, in terms of thinking about what a healthy lifestyle is." ***(Iris, 23 months postpartum)***
	Healthy and balanced diet	• "Eating healthily. Um, so lots of fresh fruit and veggies and, you know, balanced diet, like yeah, meat, and carbs, and things like that. And yeah, I suppose reasonable portions, um, but having the odd treat as well." ***(Maeve, 1 months postpartum)***<br>• A healthy diet would be, to me... we follow predominantly a plant-based diet with a lot of fish and a little bit of meat, as well. But mainly lots of vegetables, lots of fruit and fresh produce. We, um... I do pretty much all the cooking myself. And we don't eat a lot of take away." ***(Adele, 23 months postpartum)***
	Exercise and being physically active	• "It's definitely exercising.... If you do more and you are up and running more … that's what makes you feel more energetic. ***(Cara, 21 months postpartum)***<br>• "A lifestyle that, um, enables you to get out and about and, um, get some form of physical activity, um, whether it's as simple as stretching or walking." ***(Iris, 23 months postpartum)***
	Good quality sleep	• "Sleep is the most important thing. A good nice sleep basically can make you a complete different new person for the first two hours during the day." ***(Cara, 21 months postpartum)***<br>• "Obviously, um, sleeping is a really important for a healthy lifestyle. Um, and wellbeing as well. Because if you're not sleeping well, you're not gonna eat well ... it's gonna effect all elements of your life." ***(Adele, 23 months postpartum)***
	Mentally healthy and happy	• "Yeah, it's definitely the mental, um, resilience and, and being in a state of mind that, I'm relaxed and not worried about things. So, that mental state is quite important. … When I'm not anxious about something, generally its, its, yeah, uh, a healthy lifestyle is much easier." ***(Cheng, 23 months postpartum)***<br>• "Addressing mental health issues, um, related to my first pregnancy, um, has helped me come out the other end and have a healthier, healthier lifestyle." ***(Emma, 18 months postpartum)***

	Social connection	• "It's not just healthy eating or healthy weight. It's also about, um, living with healthy relationships, um, doing some things that are proactive and positive in your life." ***(Evelyn, 15 months postpartum)***<br>• "It's more than just... what you look like. Or how heavy you are. It's about your- your sense of self and um, how you relate to other people as well." ***(Patricia, 1 months postpartum)***
	Health is achieved with balance	• "I think that a, a healthy lifestyle incorporates, uh, multiple aspects, … I suppose, just a balanced life." ***(Farah, 13 months postpartum)***<br>• "Well, a healthy lifestyle for me would mean a well-balanced lifestyle, which is a combination of good, healthy food and... exercise, as well as, um, you know, opportunities to promote mental health." ***(Adele, 23 months postpartum)***<br>• "The healthy lifestyle is also having the balance between work and, and um, home life or you know, social life. Um, yeah. So yeah, not feeling like work's taking over." ***(Maeve, 1 month postpartum)***
**Barriers**		
	**THEME 2: Mentally exhausted: Lack of sleep and mental burden**	
**CAPABILTY (Physical and Psychological)**	Recovery following child-birth	• "The challenge um, and I mean, I guess now, well my body uh, physically I'm back to where I was. … So, just, you know, dealing with picking them up or things each day, um, yeah can, can be quite wearing." ***(Amelia, 17 months postpartum)***
	Sleep deprivation and fatigue	• "On the, the days where you've had that difficult overnight, um, period, I am far less likely to get up early to do any sort of exercise program because I've reprioritized the sleep … partly because when I'm really tired, … like, I've got a shorter temper, I'm just far less tolerant, I'll eat food that isn't, you know, necessarily a good choice cause you're just sort of tired and you're looking for sugar or you might have more coffees than you should." ***(Phillipa, 19 months postpartum)***<br>• "If you're feeling sleep deprived or if you're feeling, um, you know, maybe, you're really stressed or you're not getting enough time to, to do everything I think that exercise can, um, be the last priority." ***(Sophia, 1 months postpartum)***
	Mental exhaustion, cognitive overload and mental health	• "After having [my baby] and, um, uh, I guess for the, after the first six months when he was having sleep problems, um, I developed, um, postnatal anxiety... I had trouble sleeping. … I just couldn't function." ***(Sophia, 1 months postpartum)***<br>• "If things get really stressful we... sometimes we'll get more take away than I would like, or ... have to used more processed foods … you know, ... chicken nuggets from the freezer if I'm running too late from work … If things are a bit stressful, usually... yeah, exercise will go first, and then probably the mindfulness, and then probably the diet." ***(Adele, 23 months postpartum)***
	Snacking to manage fatigue	• "I know when I'm busy… and when I'm tired, then I tend to eat, I snack more." ***(Emma, 18 months postpartum)***
	**THEME 3: Time poor: Too busy to be healthy**	

	Busyness and lack of time	• "So, the main, our main issue to, you know, achieving optimal health and wellbeing is really time." ***(Adele, 23 months postpartum)***
	Navigating work and motherhood	• "I don't think work promotes healthy lifestyle." ***(Hua, 19 months postpartum)***<br>• "But working part time and that kind of, um, rushing out the door in the morning to get to work and getting home in the evening ... And then that kind of bath and bed and dinner and things routine, … it's kind of doesn't leave a lot of time... Yeah, I find it challenging to fit in exercise." ***(Georgina, 19 months postpartum)***<br>• "If it's a particularly busy week or there's a child home from day care or, you know, and you're trying to prioritize ... getting your work done, exercise will certainly be the thing that slips or get pushed to the bottom of the priority list." ***(Sophia, 1 months postpartum)***<br>• "It's a bit more of a challenge because on workdays there's literally like no time for anything extra, like it's just work and then, um, looking after [my child], then dinner, then bed… those days are just intense." ***(Maeve, 1 month postpartum)***
	Navigating demands of motherhood	• "You know, sometimes you're so busy with kids and running around after them and taking care of them that you forget all about yourself. Um, and you don't even have time to stop and think about what you're actually doing or what...might be missing in your life…. It's just that time you need that time." ***(Farah, 13 months postpartum)***<br>• "Breastfeeding has been the biggest barrier to self-care." ***(Patricia, 1 months postpartum)***
	Lack of time means unable to attend health programs, exercise or may skip meals	• "With the two kids and a family, it's really hard to find that period of time, like a block of time of very good an hour you can actually go out and jogging and then come back." ***(Cara, 21 months postpartum)***<br>• "In an ideal world, you know, I would like to exercise four or five times a week. But unfortunately given the kids and working and multiple commitments and having a husband that's not home very often, so... I don't have a lot of time to exercise." ***(Adele, 23 months postpartum)***<br>• "I haven't really had time to do any exercise as much for myself, like pretty much since he was born." ***(Frances, 13 months postpartum)***<br>• "My diet is not that great because sometimes I will go the whole day without eating anything and then I'll just have a big meal at the end of the day, just 'cause, you know, you get busy working." ***(Farah, 13 months postpartum)***
	Limited planning	• "But sometimes I'll be missing ingredients and I'll have to, you know, I can't go to the shop because I have to take [my children] with me. So, (laughs) I'll just...skip it altogether…. those are the little things that you don't really think about, that get in the way." ***(Farah, 13 months postpartum)***
**OPPORTUNITY (Social)**	**THEME 4: Unsupported: Lack of practical and social support from partner, family and friends**	
	Lack of support from partner	• "So, living with a shift worker, I find prevents some things because our life, is really revolving around his shift work and so he doesn't have a set roster. So, I never quite know when he's going to be free to look after [my child], so that I can go and take care of myself. So, I find that sometimes I just don't make plans." ***(Charlotte, 18 months postpartum)***

		• “And sort of, um, I guess fatigue after a while. You just get sick of eating your own food and cooking your own meals. Cause unfortunately we're not in a household where it's a shared responsibility.” ***(Adele, 23 months postpartum)***
	Lack of practical support from family	• “It would be nice to probably have like a little bit more, … like support from grandparents… so sometimes it doesn’t feel like a lot of it is on me.” ***(Kate, 17 months postpartum)***
	Lack of peer support *(and access to mother’s group)*	• “I’m pretty sure the [Mother’s] group wasn’t offered to me for my second or third child … and I remember when I went to the first one there was a lady there who had a third baby and she said during the introductions, ‘*I know this is my third child but I begged the nurse to let me come*.’” ***(Sophia, 1 months postpartum)***
	Social isolation *(especially when family is interstate or overseas)*	• “I didn't grow up here, I didn't study here, so my friends are [overseas], so I don't have that support here.” ***(Alice, 15 months postpartum)***<br>• “We're not in a situation where we've got any family or friends or anybody in Melbourne that we can call on for babysitting or child care.” ***(Georgina, 19 months postpartum)***
**OPPORTUNITY (Physical)**	**See Barriers and Enablers Table** ***(not reported as a theme)***	
	Costs and inadequate finances	• “When you're at home on maternity leave and you already have a reduced salary, any additional expenses are something that you try to avoid as best you can. Like we, we barely got through, I was only on maternity leave for eight months … and I ended up returning to work because we just can't afford it.” ***(Evelyn, 15 months postpartum)***<br>• “I mean the closest shop to me is completely unaffordable. It's an organic grocer that's, um, very expensive.” ***(Adele, 23 months postpartum)***
	Unsupportive environments for activity (*including weather*)	• “It is much harder to create opportunities for movement when you're only allowed out for short periods a day and it’s, you know, often weather dependent.” ***(Phillipa, 19 months postpartum)***
**MOTIVATION (Automatic and Reflexive)**	**THEME 5: A balancing act: Difficulty prioritising self in the midst of competing priorities**	
	Difficulty prioritising self	• “Cause I'm a single mum, so, um, it's really hard to get any time on my own to exercise…. Um, and that's something I really enjoy, but that's a challenge.” ***(Emma, 18 months postpartum)***<br>• “Cause I certainly think, um, uh, certainly in the early years when... Or at the early, um, time when my first daughter was born that I definitely didn't prioritize my own, um, kind of wellbeing and exercise or physical activity very much at all.” ***(Georgina, 19 months postpartum)***
	Feeling unmotivated	• “I do feel less motivated to do it because sometimes even if these only take about 30 minutes... you've got to push yourself hard sometimes to keep doing it.” ***(Cara, 21 months postpartum)***<br>• “I'm sort of in one of those like lulls where I'm just not in the mood. Probably the start of winter, and I'm just not focused on it.” ***(Kate, 17 months postpartum)***
	Prioritise children’s and family’s needs	• “By the time I get home, I'm basically uh, feeding [my child] and putting him to bed and then we get our own dinner and then usually, I'm in bed not soon after.” ***(Eloise, 14 months postpartum)***

		• "You have a lot on your plate, a lot of things you're juggling, like, you know, you've got a job and you're also running a house, and you're also the primary carer for two people… it takes a lot of mental energy to make sure you've got everything you need and being organized." ***(Kate, 17 months postpartum)***
**Facilitators**		
	**THEME 6: Fitting it in: organise and plan ahead**	
**CAPABILITY (Physical and Psychological)**	Organising and planning ahead	• "When the little one has his nap, sometimes I'm thinking, should I do my exercise now or should I go out and wash the clothes or, you know, clean these and wash dishes and clean." ***(Olivia, 8 months postpartum)***<br>• "It makes me feel better knowing, okay, Mondays I do this, Tuesdays, I do this... it let's me sort of be organized and prepared and help shape the week." ***(Kate, 17 months postpartum)***
	Integrating healthy behaviours into routine where possible	• "Kind of depends on her wake-up time. If she wakes up early enough, we squeeze in a morning walk." ***(Maeve, 1 month postpartum)***<br>• "So, I, I train home and then do 15 minutes and get [my child] from child care … If I can squeeze in um, three 15-minute bicycle sessions a, a week, during the week days, that's very good." ***(Cheng, 23 months postpartum)***<br>• "We try not to use the car, because we don't have to, so I kind of walk and... or bike everywhere." ***(Emma, 18 months postpartum)***
	Consistent and regular routines	• "For me trying to sort of focus on maintaining, you know, health and wellbeing and also just a bit of happiness and, you know, that, a routine is quite important… I'm probably maybe sometimes a little bit too rigid just on myself, but I, it makes me feel better." ***(Kate, 17 months postpartum)***<br>• "If it's something that fits into your regular routine, so if you're seeing those mothers on a regular basis and it's something that you could implement." ***(Phillipa, 19 months postpartum)***
	Prioritising healthy behaviours	• "I do try and prioritize getting to bed at a reasonable time, because I know that if I'm well rested, everything else is easier." ***(Emma, 18 months postpartum)***<br>• "So, making the time and prioritizing exercise helps." ***(Adele, 23 months postpartum)***
	Creating time and space for self	• "You really need to take care of your mental health and you need to make sure that you, you have your space." ***(Farah, 13 months postpartum)***<br>• "It's just about saying it's okay to have some time to yourself, whether it's with friends or going for a walk by yourself, rather than feeling guilty that you've left your son behind." ***(Eloise, 14 months postpartum)***
	**THEME 7: Friends for health: the importance of other mothers**	
**OPPORTUNITY (Social)**	Modelling, accountability, and encouragement from peers	• "They were posting pictures on what they see when they went for a walk. So, oh yeah, … it actually is pushing me to continue to do it. And in a way I feel, I feel quite good after that." ***(Olivia, 8 months postpartum)***

	Exercising with others is helpful	• "And I don't think I would've had the courage to sign up, do it on my own. … So yeah, the fact that we did it as a group, I think made the big difference." ***(Evelyn, 15 months postpartum)***<br>• "The days that I was with [my baby], I'd like, go to the park or go for a walk with another mom or something. So yeah, that was good for like getting out of the house and getting some exercise and also talking to other, other mom's with similar age babies." ***(Frances, 13 months postpartum)***
	Social Connection - mothers and church support groups are beneficial	• "I think we've got a fairly big social connection with our church. So, uh, I'm quite involved with my church and there are groups there that really helped me to, get out of the house and ... connect with other people." ***(Charlotte, 18 months postpartum)***
	**THEME 8: The better half: partners as source of practical and moral support for health**	
	Practical support from partners	• "When he's around more like on the weekends and, and stuff like that, then he can take over the babysitting duties." ***(Farah, 13 months postpartum)***<br>• "I feel comfortable with saying to my husband, look, can you take our son on Saturday so that I can get out of the house and go catch up with a friend." ***(Evelyn, 15 months postpartum)***<br>• "When my mom is not here, what I've found is um that's when the husband plays a very important role here. So, if he can actually take care of the girls and that I managed to have sneak out for an hour or two, for, um, just sort of self-care, just to have that mental break." ***(Cara, 21 months postpartum)***
	Partners with similar health goals	• "I think it's, uh, it's very important that your partner is, um, invested in, um, having a healthy lifestyle, um, with the family. Um, if, if you're both not in it together, um, it's just going to be a big struggle." ***(Emma, 18 months postpartum)***<br>• "I'm fortunate in that, you know, my partner is sort of recognizes the value that [exercise] is for me, even though it might be different for him… we both sort of acknowledge that the way we need to do exercise is different, but it's still important." ***(Phillipa, 19 months postpartum)***
	**THEME 9: It takes a village: practical help from extended family**	
	Practical support from family	• "My sister's been a great help … because she only lives around the corner … so just having, you know, that, that support within the family has been great." ***(Evelyn, 15 months postpartum)***<br>• "My youngest sister stayed with us, um, for a couple of weeks … so yeah, she was great to have just that second pair of hands to go, oh look, I'll feed them, I'll, you know, I'll put them sleep, I'll do that. So, um, my sister and my parents and [my partner's] parents, [my partner], or particularly my mum and [my partner's] mother have been really helpful too." ***(Amelia, 17 months postpartum)***
	Assistance with babysitting and childcare	• "My mum can do some babysitting." ***(Maeve, 1 month postpartum)***<br>• "When my mum's here, it makes it easier that when you want to have a little bit of break with the kids and you can sort of say, "mum, could you please help?" and because she lives with us, um, she will be able to just sort of, and she's

		also very hands on. So, she can just take care of the girls and if I want to go out to just have some self-time and also my husband and I want to have a date night, we can actually do that." ***(Cara, 21 months postpartum)***
**OPPORTUNITY (Physical)**	**See Barriers and Enablers Table** ***(not reported as a theme)***	
	Adequate finances	• "I also think we're a bit lucky in that, you know, we're both working, we've got the opportunity to go to the shops and buy food that is healthy … We can buy fresh veggies and we can do all of those things. So, we're not constrained by, um, you know, the financial costs of being healthy." ***(Phillipa, 19 months postpartum)***<br>• "I'm not saying we're billionaires, but being able to afford a healthy lifestyle makes a big difference. So, we can afford to buy good fruit and vegetables and that's what we prioritize our spending on, is, um, good produce." ***(Adele, 23 months postpartum)***
	Supportive environment for physical activity and healthy food choices	• "We live in, um, like in nice area… so, when you're having a walk, it's very relaxing and it's got lots of, um, parks and gardens that contribute." ***(Cara, 21 months postpartum)***<br>• "We get fresh food delivered to home, which makes it, like, … easier." ***(Iris, 23 months postpartum)***
	Supportive work environment and work-life balance	• "We actually I have a really good balance. So, my husband is self-employed … and as a result, he can be quite flexible with his hours or work from home when he needs to. So, we find that we're able to juggle that quite easily." ***(Evelyn, 15 months postpartum)***<br>• "I do find when I actually go to work I enjoy my days a lot more feeling very productive and um, almost every afternoon when I go to pick up the kids, it almost feels I can't wait to see them." ***(Cara, 21 months postpartum)***
**MOTIVATION**	**THEME 10: Reasons to engage: motivators for healthy behaviours**	
	Enjoyment of cooking and exercise	• "In terms of exercise, acknowledging that it's not sort of a one size fits all and everyone likes to do something different. So, it's a matter of just finding the thing you like and, and leaning into that." ***(Phillipa, 19 months postpartum)***<br>• "I'm quite a good cook. So that definitely helps. Because I can make healthy food pretty easily without any, um, issues... For me, cooking's actually a way to switch off, and, um, tune out. So that's kind of my relaxation time." ***(Adele, 23 months postpartum)***
	Exercise to improve mental health	• "I had very bad depression with [my first child] but then I was okay with [my second child] .... I think going to the gym really helped." ***(Hua, 19 months postpartum)***<br>• "So, the Pilates that I do... I think that's not only from a perspective of physical health, but I think that's also a, um, kind of a mental health perspective." ***(Georgina, 19 months postpartum)***
	Motivating self-talk	• "Just trying to keep, trying to remember that I'll feel better once it's done." ***(Phillipa, 19 months postpartum)***<br>• "I mean, sometimes when you are tired, you know that you feel good once you've been out and done a big walk together. So, you just kind of get out and go through it anyway." ***(Charlotte, 18 months postpartum)***
	Goal setting	• "It's kind of always a mission of mine to at least go for a daily walk, um, even if I'm working from home." ***(Maeve, 1 month postpartum)***

Table S4. Mapping of Capability, Opportunity and Motivation (COM) behaviour model domains to intervention functions as per Michie, Atkins and West [13].

**COM-B Domains**	**TDF Domains**	**Intervention functions**								
		**Education**	**Persuasion**	**Incentivisation**	**Coercion**	**Training**	**Restriction**	**Environmental Restructuring**	**Modelling**	**Enablement**
Physical Capability	Physical Skills									
Psychological Capability	Knowledge									
	Cognitive and Interpersonal Skills									
	Memory, Attention and Decision Processes									
	Behavioural Regulation									
Physical Opportunity	Environmental context and resources									
Social Opportunity	Social influences									
Reflexive Motivation	Professional/social role and identity									
	Beliefs about capabilities									
	Optimism									
	Beliefs about consequences									
	Intentions									
	Goals									
Automatic Motivation	Reinforcement									
	Emotion									

Legend: Capability, Opportunity, Motivation Model of Behaviour Change (COM-B), Theoretical Domains Framework (TDF).

Table S5. Mapping of intervention functions to behaviour change techniques as per Michie, Atkins and West [13].

Intervention function	Most frequently used Behaviour Change Techniques
**Education**	• Feedback on behaviour<br>• Feedback of outcome of behaviour<br>• Monitoring of behaviour by others without evidence of feedback<br>• Monitoring of outcome of behaviour by others without evidence of feedback<br>• Self-monitoring of behaviour
**Persuasion**	• Demonstration of the behaviour<br>• Instruction on how to perform a behaviour<br>• Feedback on outcome of behaviour<br>• Self-monitoring of behaviour<br>• Behavioural practice/rehearsal
**Incentivisation**	• Feedback on behaviour<br>• Feedback of outcome of behaviour<br>• Monitoring of behaviour by others without evidence of feedback<br>• Monitoring of outcome of behaviour by others without evidence of feedback<br>• Self-monitoring of behaviour
**Coercion**	• Feedback on behaviour<br>• Feedback of outcome of behaviour<br>• Monitoring of behaviour by others without evidence of feedback<br>• Monitoring of outcome of behaviour by others without evidence of feedback<br>• Self-monitoring of behaviour
**Training**	• Demonstration of the behaviour<br>• Instruction on how to perform a behaviour<br>• Feedback on behaviour<br>• Feedback of outcome of behaviour<br>• Self-monitoring of behaviour<br>• Behavioural practice/rehearsal
**Restriction**	*No BCT linked to this as BCTs are focused on the individual not the external environment*
**Environmental Restructuring**	• Adding objects to the environment<br>• Prompts/cues<br>• Restructuring the physical environment
**Modelling**	• Demonstration of the behaviour
**Enablement**	• Social support (unspecified)<br>• Social support (practical)<br>• Goal setting (behaviour)<br>• Goal setting (outcome)<br>• Adding objects to the environment<br>• Problem solving<br>• Action planning<br>• Self-monitoring of behaviour<br>• Restructuring the physical environment<br>• Review behaviour goal<br>• Review outcome goal

References

13. Michie, S.; Atkins, L.; West, R. *The Behaviour Change Wheel: A Guide to Designing Interventions*; Silverback Publishing: Surrey, UK, 2014; pp. 1–329.

19. Tong, A.; Sainsbury, P.; Craig, J. Consolidated criteria for reporting qualitative research (COREQ): A 32-item checklist for interviews and focus groups. *Int. J. Qual. Health Care J. Int. Soc. Qual. Health Care* **2007**, *19*, 349–357.
